# Indoor bacterial and fungal aerosols as predictors of lower respiratory tract infections among under-five children in Ibadan, Nigeria

**DOI:** 10.1186/s12890-022-02271-w

**Published:** 2022-12-09

**Authors:** Adekunle Gregory Fakunle, Nkosana Jafta, Lidwien A. M. Smit, Rajen N. Naidoo

**Affiliations:** 1 Discipline of Occupational and Environmental Health, University of KwaZulu-Natal, 321 George Campbell Building Howard College Campus, Durban, 4041 South Africa; 2 Department of Public Health, College of Health Sciences, Osun State University, Osogbo, Osun State Nigeria; 3 Institute for Risk Assessment (IRAS), Utrecht University, Utrecht, The Netherlands

**Keywords:** Indoor microbial aerosols, Lower respiratory tract infections, Under-five children, Sub-Saharan Africa, Ibadan

## Abstract

**Background:**

This study aimed to investigate the association between exposure to diverse indoor microbial aerosols and lower respiratory tract infections (LRTI) among children aged 1 to 59 months in Ibadan, Nigeria.

**Methods:**

One hundred and seventy-eight (178) hospital-based LRTI cases among under-five children were matched for age (± 3 months), sex and geographical location with 180 community-based controls (under-five children without LRTI). Following consent from caregivers of eligible participants, a child’s health questionnaire, clinical proforma and standardized home-walkthrough checklist were used to collect data. Participant homes were visited and sampled for indoor microbial exposures using active sampling approach by Anderson sampler. Indoor microbial count (IMC), total bacterial count (TBC), and total fungal count (TFC) were estimated and dichotomized into high (> median) and low (≤ median) exposures. Alpha diversity measures including richness (*R*), Shannon (*H*) and Simpson (*D*) indices were also estimated. Conditional logistic regression models were used to test association between exposure to indoor microbial aerosols and LRTI risk among under-five children.

**Results:**

Significantly higher bacterial and fungal diversities were found in homes of cases (*R* = 3.00; *H* = 1.04; *D* = 2.67 and *R* = 2.56; *H* = 0.82; *D* = 2.33) than homes of controls (*R* = 2.00; *H* = 0.64; *D* = 1.80 and *R* = 1.89; *H* = 0.55; *D* = 1.88) p < 0.001, respectively. In the multivariate models, higher categories of exposure to IMC (aOR = 2.67, 95% CI 1.44–4.97), TBC (aOR = 2.51, 95% CI 1.36–4.65), TFC (aOR = 2.75, 95% CI 1.54–4.89), bacterial diversity (aOR = 1.87, 95% CI 1.08–3.24) and fungal diversity (aOR = 3.00, 95% CI 1.55–5.79) were independently associated with LRTI risk among under-five children.

**Conclusions:**

This study suggests an increased risk of LRTI when children under the age of five years are exposed to high levels of indoor microbial aerosols.

**Supplementary Information:**

The online version contains supplementary material available at 10.1186/s12890-022-02271-w.

## Introduction

The microbiota of the indoor environment is an assemblage of a wide range of microorganisms including bacteria, fungi, viruses, archaea, and protists [1, 2]. These microorganisms occur ubiquitously even within indoor environments such as dwellings where humans, including children under the age of five years, spend more than 90% of their time [2, 3]. Therefore, understanding the processes and features that structure the microbiota within the indoor environment may provide clues to improve children’s health. The increasing interest in the microbiota of the built environment is largely due to the wider recognition that exposures to microbes within the residential indoor environment are associated with a vast number of adverse health outcomes, including infectious diseases such as lower respiratory tract infections (LRTIs), allergies and cancer [2]. In addition, studies suggest that certain indoor microbial communities can have beneficial health impacts, which has sparked interest in how to shape the indoor microbiota and design effective interventions [4].

Determining the risk factors for LRTI in infants and young children is important because LRTIs including pneumonia and bronchitis contributes largely to the burden of childhood morbidity and mortality [5], therefore posing a major public health challenge in sub-Saharan Africa [6]. An estimated 921,000 children died of LRTI in 2015, and more than 95% of these deaths occur in low-and-middle income countries (LMIC) predominantly South Asia and sub-Saharan Africa [7, 8]. In 2017 community-acquired LRTI was the single largest cause of under-five mortality, accounting for 140,520 (19%) deaths in Nigeria [9]. This number is the highest in Africa and the third highest estimated number of childhood death from LRTI in the world [10].

Several epidemiological studies have reported a strong relationship between occupant density, human activity and microbial burden within the indoor environment [11, 12]. Due to this association, under-five children are more likely at risk of LRTIs because they spend a considerable proportion of time in the indoor environment during a period of intense growth and development of the immunologic and respiratory systems [13]. Unraveling exposure–response relationships between the indoor microbial aerosols and LRTI among under-five children is an important step to disease prevention. Despite the increasing evidence that microbial diversity might contribute stronger to health effects than the abundance of single components [14, 15], the majority of the available studies focused on individual microbial entities. A recent systematic review [16] showed that the majority of studies that aimed at investigating the association between exposure to indoor bacterial and/or fungal communities and LRTI have used proxy measures such as self-reported presence of visible moulds [17, 18], while only very few have attempted to quantify microbial exposures [19, 20].

Despite the high burden of LRTI among under-five children in LMIC, there is a paucity of studies exploring exposure–response relationships between indoor microbial communities and LRTI, especially in sub-Saharan Africa. Therefore, this study investigated the association between exposure to diverse indoor microbial aerosols and lower respiratory tract infections (LRTI) among children aged 1 to 59 months in Ibadan, Nigeria.

## Materials and methods

### Study setting and design

The study was carried out in Ibadan which is the third largest city in Nigeria in terms of population density with a total population of over 6 million people within metropolitan area [21]. LRTI cases (n = 178) were recruited from three health facilities in Ibadan viz: Otunba-Tunwase Children Emergency Clinic of the University College Hospital (a 500-bed tertiary health institution and a major referral health centre in Southwest Nigeria), Ade-Oyo Maternity Teaching Hospital (a state-owned general hospital mostly patronized by residents of Ibadan, especially those from low and middle socioeconomic status); and Oni-memorial Children Hospital (a secondary health institution that provides health care services exclusively for children 12 years of age and below). Eligible controls (n = 180) were identified from the same community as the cases and recruited after obtaining consent. These communities, all located in Ibadan, represented the 11 local government areas in Ibadan with majority of cases and controls from Bodija, Apete, Apata, Agbowo, Olomi, Ashi, Yemetu, Oluyole, and Oke-Ado. Most of these communities are semi-urban with medium density residential areas inhabited majorly by the Yoruba speaking population comprising individuals and households in the low and middle socioeconomic class.

### Definition and selection of cases and controls

The study employed a case–control design, where cases (under-five children with LRTI) were age (± 3 months), sex and geographical location matched to controls (under-five children without LRTI). The details of the study protocol, and recruitment procedures have been published elsewhere [22].

At the presenting hospital, children were screened and those who met the eligibility criteria (< 5 years of age; admitted for LRTI; and residing in Ibadan), and whose caregivers gave consent, were included in the study. Diagnosis of LRTI in children was carried out by a doctor based on chest radiography including the presence of one or more of the chest radiographic features of patchy, segmental, or lobar consolidation; +/− a positive air bronchogram; and +/− pleural effusion. Matchable controls were primarily recruited from the same community where the cases reside during follow-up of cases. The approach used in the identification and recruitment of community controls was that the caregivers of recruited cases were asked to identify a neighbour whose child is of the same age group and sex as the case. Then, screening of potential controls at the community was performed by the same doctor that assessed the cases for the presence/absence of respiratory signs and symptoms. Therefore, for every case of LRTI recruited from the hospitals, an age/sex matched control was identified, assessed by a doctor, and confirmed to not have LRTI and/or any of the respiratory signs (such as fast breathing, respiratory crackles, intercostal recession, and lower chest wall indrawing) and symptoms (such as cough, fever, wheezing and difficulty in breathing) in the past 30 days [20, 23]. We chose this extended period of no signs or symptoms to ensure that controls were not likely to be in the pre-clinical, asymptomatic stage of an acute infection.

Cases with other systemic illnesses such as measles, symptomatic congenital heart disease, congenital malformation, or Acquired Immune Deficiency Syndrome (AIDS) and those that presented symptoms of measles or pertussis in the preceding 10 days were excluded from the study at the data collection phase to avoid outcome definition bias. Similarly, controls hospitalized for respiratory or allergic conditions or with complaints of an LRTI in the past 30 days were excluded from the study.

### Data collection and procedure

The caregivers of recruited cases and controls were interviewed by trained public health personnel using a structured questionnaire modified from a previously validated child health questionnaire to obtain basic demographic, household and family characteristics [24]. In addition, the health status of the child was determined by a trained nurse using a clinical proforma.

### Clinical and health data

A clinical proforma (Additional file 1: Table S1) was used to collect vital health information about the child such as breastfeeding status/duration, immunization status/vaccine received, anthropometric measurements, respiratory symptoms/signs, severity of LRTI/other diagnosis, x-ray findings, and outcome of hospital admission.

### Home walkthrough assessments

After enrollment, a relative of cases led the study team to their homes within 24 h for home survey and environmental monitoring during which a matchable control from within the same community as the case was identified and recruited. A validated walkthrough checklist [25] was used by trained inspectors to document real-time observations on housing conditions (including type of house, material used in the construction of roof, walls and floor, presence of doors and windows, visible mould growth and dampness or moisture on surfaces) and household characteristics (such as house ownership, type of cooking and heating energy sources used, number of rooms, occupancy, number of tobacco smokers, and keeping of pets). The cooking and heating fuels used in the households were classified as clean (electricity and liquid petroleum gas (LPG)) and dirty (kerosene and wood) and those households that used a combination of clean and dirty fuels were classified as mixed fuel [25]. Occupant density was defined as the total number of occupants divided by the total number of rooms present. The presence of visible water stain/dampness was defined as any sign of moisture damage on the roof, walls or housing materials at the participating child’s sleeping area while visible mould growth was said to be the presence of mould growth on moist surfaces within the house or the smell of mouldy odour.

### Indoor environmental monitoring and microbial estimation

#### Indoor environmental conditions

Indoor air temperature and relative humidity (RH) were monitored in homes of recruited cases and controls using EXTECH datalogger model 42270. The datalogger was installed in the homes of cases and controls for 24 h, and the readings were retrieved using TRLog software version 4.0 (FLIR Commercial Systems Inc., TownsendWest, Nashua, NH, USA).

### Air sampling and microbiological analysis

The concentration of indoor air bacteria and fungi was estimated by collecting air samples in homes of cases and controls using a BioStage Anderson air sampler equipped with 90 mm petri dish containing agar medium [Nutrient, Blood, and McConkey Agar for bacteria isolates and Saboraud Dextrose Agar (SDA) for fungi isolates] prepared according to the manufacturer’s specifications. All samples were collected within 24 h after identification and recruitment of the participants. Samples were collected in the daytime at about 1.5 m height (to simulate the location of the breathing zone) in the room where the child sleeps/plays, at an air flow rate of 28.3 ± 2 l/min for 15 min [26]. Collected samples were then stored in an ice pack and transferred to the laboratory within 24 h before incubation. Cultures on Nutrient, Blood, and McConkey Agar were incubated using a microbiological incubator at 35 ± 2ºC for 48 h while SDA plates were incubated at room temperatures for 5 days with daily observation. The number of colonies on all agar media were counted using a Quebec darkfield colony counter (Cambridge Instruments, Inc., Buffalo, NY). The total bacterial count (TBC) and total fungal count (TFC) were estimated as colony-forming units per cubic metre (cfu/m^3^) using the formula [27]:

1 $${\text{Total\,bacterial/fungal\,count}}\,\left( {{\text{cfu/m}}^{{3}} } \right) = \frac{{[{\text{Total\,bacterial/fungal\,colonies}} \times 10^{3} ]}}{{\left[ {\text{Air\,flow\,rate}} \times {\text{time\,(minutes)}} \right]}}$$

Afterwards, the identification and classification of colonies was performed to obtain distinct specie of the organisms. The identification of bacterial isolates were carried out based on their colonial morphology, cellular morphology and biochemical characteristics [28]. The fungal isolates were characterized and identified based on their macroscopic and microscopic characteristics [29]. The indoor microbial count (IMC) was estimated based on the mathematical model below:

2 $${\text{Indoor\,Microbial\,count:\,IMC}}\,\left( {{\text{cfu/m}}^{{3}} } \right) = \mathop \sum \limits_{i = 1}^{k} mi = m_{{}} + m_{2} + \cdots + m_{k}$$

where “M” is the total count (cfu/m^3^) for the individual bacterial and fungal species “*i*” and “k” is the last bacterial/fungal species identified.

### Microbial alpha-diversity measures

Exposure to indoor microbial aerosols in this study was defined in terms of the IMC and the microbial alpha-diversity indices as described below.

The microbial richness (*R*), Shannon diversity index (*H*) and Simpson’s diversity index (*D*), were estimated based on the individual distinct species of bacteria and fungi identified representing distinct species. Species richness, *R* defined as the number of species per sample was estimated using the formula:

3 $${\text{Species\,richness}}\,(R) = {\text{n/N}}$$

where ‘n’ is the number of species and ‘N’ is the number of individual microorganisms in the sample.

The Shannon diversity index, *H,* was used as a measure of species evenness. Species evenness refers to how close in numbers each species in a sample is.

4 $${\text{Shannon's\,diversity\,index}}\,(H) = \mathop \sum \limits_{i = 1}^{k} pi \left( {In\,pi} \right)$$

where p is the proportion (n/N) of individuals of one particular species found (n) divided by the total number of species found (N), “*ln”* is the natural log, and *k* is the number of species.

To obtain the Simpson's diversity index (*D*), the proportion of bacterial and fungal species “*i”* relative to the total number of bacteria and fungi genera (*p*_*i*_) was calculated and squared. The squared proportions for all the microbial species were summed, and the reciprocal taken as indicated in Eq. 4 below:

5 $${\text{Simpson's\,diversity\,index}}\,(D) = \frac{1}{{\sum_{i = 1}^{k} pi^{2} }}$$

where “*i”* is the proportion of bacterial and fungal species and (*p*_*i*_) is the total number of bacteria and fungi genera.

### Statistical analysis

All data from completed child health questionnaires, clinical proforma, household walkthrough checklists and indoor air sample results were entered into Excel spreadsheets. Data were subjected to logic checks to ensure validity and consistency. A validated and complete dataset was exported to Stata SE 12.0, and R statistical program (version 4.0.0) for further analyses.

**Fig. 1:**
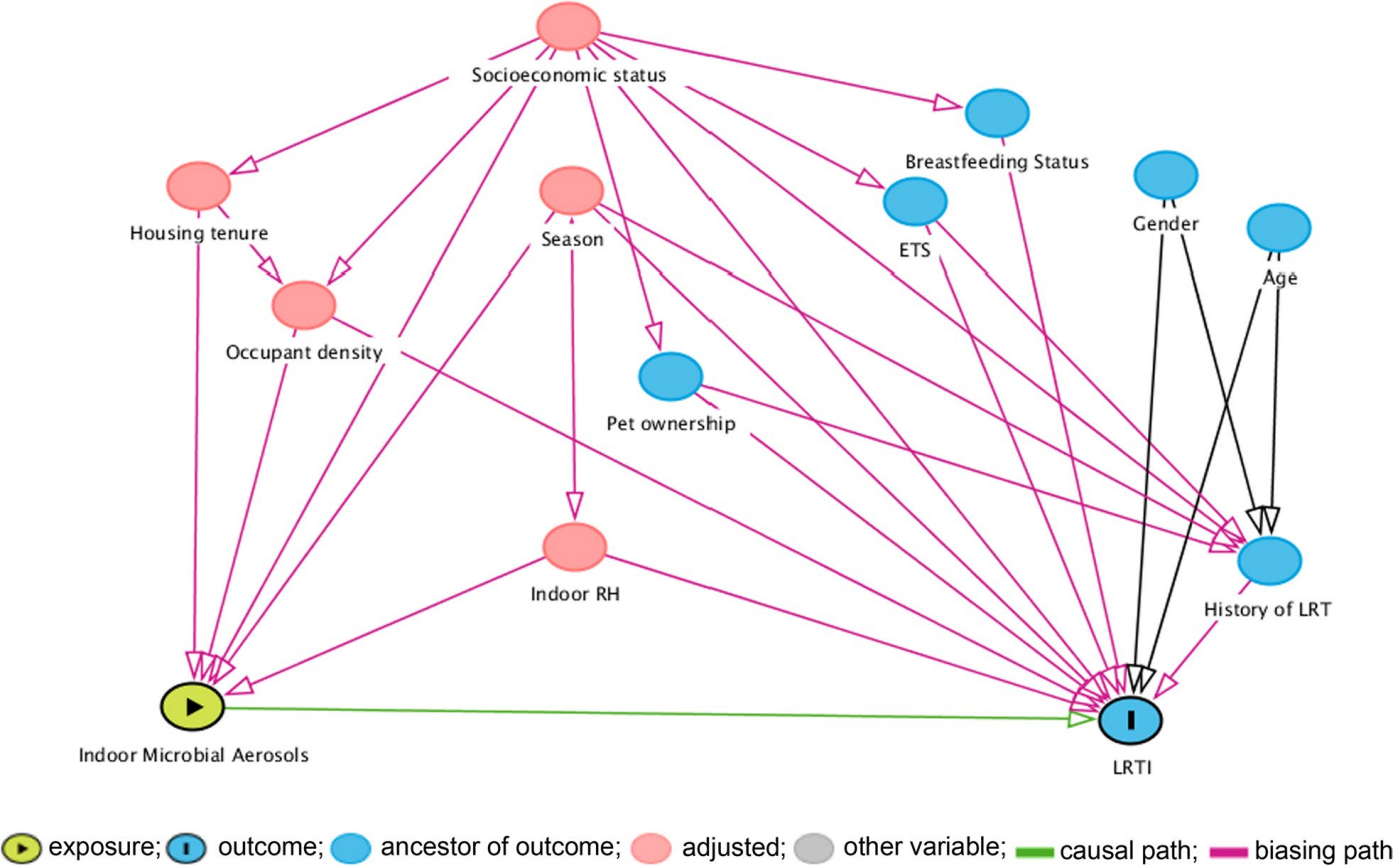
Directed acyclic graph (DAG) demonstrating causal relationships affecting the association between exposure to indoor microbial aerosols and lower respiratory tract infections (LRTI). *ETS* environmental tobacco smoke, *RH* relative humidity, DAG was created using http://www.dagitty.net/

We analyzed the associations between microbial exposures and case status using conditional logistic regression with adjustment for potential confounders that were not used in the matching. The exposure variables including microbial counts (IMC, TBC, and TFC), microbial richness and Simpson’s diversity index were dichotomized at the median to define high and low exposures. The multiple logistic regression models included covariates based on the hypothesized connection in the directed acyclic graph (DAG) (Fig. 1). The individual circle in the DAG connotes an individual exposure (node) of theoretical relevance. The association of interest is the link represented by the green arrow connecting indoor microbial aerosols and LRTI. Age and sex (blue nodes) are theoretically causally associated with the outcome alone (ancestors of outcome). The other exposure (red node) is theoretically causally associated with both the exposure and the outcome. Therefore, housing tenure, season, occupant density, keeping pets, season, history of LRTI, cooking fuel and environmental tobacco smoking were identified as variables requiring adjustment. We further assessed associations between risk factors and case status using McNemar’s test for paired categorical outcomes. We analysed differences in the distribution of IMC, TBC and TFC of cases and controls using Mann Whitney U test. Correlation between the microbial counts (IMC, TBC and TFC), alpha-diversity indices and environmental variables among cases and controls were assessed using Spearman’s rank correlation analysis.

Conditional likelihood was employed to estimate the odds ratios (ORs) and 95% confidence intervals (CIs) in eight models. All statistical tests of hypotheses are two-sided with a p value < 0.05 considered significant.

## Results

Boys (60.1%) more frequently presented with LRTI than girls. The median (IQR) age of participants was 5 (6) months (Table 1). Notably, more cases (15.7%) had a previous history of LRTI than controls (9.0%). In addition, the proportion of subjects with one or more under-five siblings was greater among cases (94.4%) than controls (86.5%) and a smaller proportion of cases (69.1%) compared to control (84.2%) were exclusively breastfed. Fewer mothers of cases had a secondary education (36.0%) compared to controls (46.1%) and the proportion of any smoker between cases and controls was significantly different (11.2% vs 5.1%; p = 0.03).

**Table 1:** Description [n (%)] of child and caregiver characteristics among cases and controls

Sociodemographic variables		Cases (n = 178); n (%)	Controls (n = 180); n (%)	p value
Child characteristics				
Sex	Male	107 (60.1)	110 (61.8)	0.74
Age (months)	Median (IQR)	5 (6)	5 (6)	0.97^†^
Number of siblings U5 years	≥ 1	168 (94.4)	154 (86.5)	0.012
	Mean ± SD	1.69 ± 0.73	1.35 ± 0.76	< 0.001
Breastfeeding	Exclusive	121 (69.1)	149 (84.2)	0.001
Immunization	Up to date	26 (14.6)	27 (15.2)	0.88
History of LRTI	Yes	28 (15.7)	16 (9.0)	0.04
Caregiver’s characteristics				
Age (years)	Mean ± SD	30.44 ± 5.16	32.88 ± 5.59	< 0.001
Marital status	Married	170 (95.5)	168 (93.3)	Ref
	Cohabiting	6 (3.4)	5 (2.8)	0.88
	Divorced/widowed	2 (1.1)	7 (3.9)	0.19
Mother’s educational status	No education	13 (7.3)	5 (2.8)	Ref
	Primary	35 (19.7)	19 (10.7)	0.57
	Secondary	64 (36.0)	84 (46.1)	0.04
	Tertiary	66 (37.1)	72 (40.4)	0.07
Father’s educational status	No education	2 (1.1)	3 (1.7)	Ref
	Primary	55 (30.9)	33 (18.3)	0.31
	Secondary	46 (25.8)	64 (35.6)	0.72
	Tertiary	75 (42.1)	80 (44.4)	0.58
Mother’s occupation	Gov. employed	51 (28.7)	53 (29.4)	Ref
	Self-employed/trading	123 (69.1)	113 (62.8)	0.86
	Farming	4 (2.2)	14 (7.8)	0.43
Mother’s monthly income ($)	> 100	33 (18.5)	46 (25.6)	Ref
	≤ 100	64 (36.0)	42 (23.3)	0.14
	Refuse to answer	81 (45.5)	92 (51.1)	
Any smoker in the house	Yes	20 (11.2)	9 (5.1)	0.03

Statistical analysis was carried out using chi-square test for categorical variables and independent sample t-test for quantitative variables; *IQR* Interquartile range; ^†^p value obtained using Mann–Whitney U

Occupant density > 5 (33.1% vs 19.1%; p = 0.01), presence of visible dampness (11.2% vs 2.2%; p < 0.001), and presence of visible moulds (21.9% vs 3.9%; p = 0.001) differed significantly in homes of cases and controls. A significant difference was observed in the use of clean cooking fuel (83.1% vs 91.6%; p = 0.02), pet ownership (38.8% vs 20.2%; p < 0.001), and house ownership (25.8% vs 41.0%; p = 0.003) between cases and controls respectively. In terms of the meteorological conditions in homes of cases and controls, the mean (SD) indoor RH (%) in homes of cases (68.02 ± 12.99%) versus controls (64.27 ± 10.58%), p = 0.003, differed significantly (Table 2).

**Table 2:** Description [n (%)] of home and exposure characteristics among cases and controls

Home characteristics		Cases (n = 178); n (%)	Controls (n = 180); n (%)	p value
Type of home	A room apartment	65 (36.5)	39 (21.9)	0.05
	Mini flat	88 (49.4)	91 (51.1)	0.52
	Apartment building/flat	8 (4.5)	23 (12.9)	0.23
	Bungalow	17 (9.6)	25 (14.0)	Ref
Total number of rooms in the house	1–3	152 (85.4)	104 (57.8)	< 0.001
	≥ 4	26 (14.6)	76 (42.2)	Ref
	Mean ± SD	2.70 ± 1.29	3.81 ± 1.79	0.002
Occupant density	≤ 2	19 (10.7)	28 (15.7)	Ref
	3–5	100 (56.2)	118 (65.2)	0.50
	> 5	59 (33.1)	34 (19.1)	0.01
	Mean ± SD	4.78 ± 2.10	4.14 ± 1.62	0.002
House ownership	Owned	46 (25.8)	73 (41.0)	Ref
	Rented	132 (74.2)	107 (59.0)	0.003
House wall construction	Wood	1 (0.6)	2 (1.1)	Ref
	Cement	147 (82.6)	164 (91.0)	0.74
	Mud	30 (16.9)	14 (7.9)	0.30
Visible water stain/dampness in CSA	Yes	20 (11.2)	4 (2.2)	0.001
Visible mold growth presence in CSA	Yes	39 (21.9)	7 (3.9)	< 0.001
Cooking area	Kitchen	131 (73.6)	152 (85.4)	Ref
	Corridor	32 (18.0)	20 (11.2)	0.06
	Where the child sleeps	15 (8.4)	6 (3.4)	0.04
Primary cooking fuel	Clean	148 (83.1)	165 (91.6)	0.02
Pet ownership	Any type	69 (38.8)	36 (20.2)	< 0.001
Season	Dry	71 (39.9)	89 (50.0)	Ref
	Wet	107 (60.1)	89 (50.0)	0.03
Indoor RH (%)	Mean ± SD	68.02 ± 12.99	64.27 ± 10.58	0.003
Indoor temperature (°C)	Mean ± SD	31.46 ± 2.21	31.07 ± 2.12	0.09

Occupant density—total no. of occupants/no. of rooms

*CSA* child’s sleeping area, *RH* relative humidity

Statistical analysis was carried out using chi-square test for categorical variables and independent sample t-test for quantitative variables

**Fig. 2:**
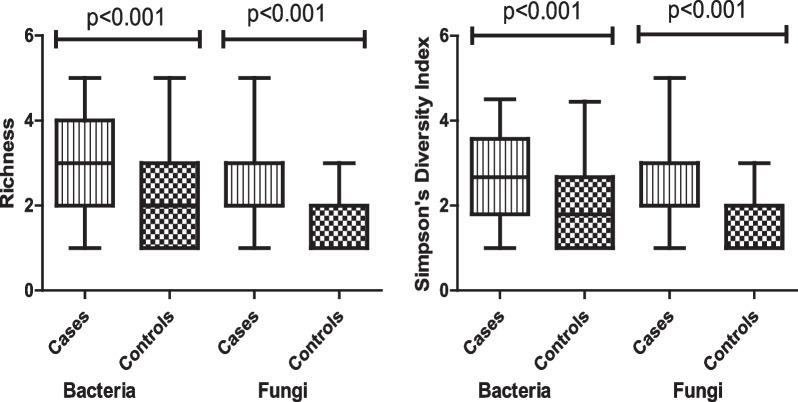
Microbial richness and diversity index in homes of under-five children

The overall median IMC, TBC, and TFC across the sampling period was 515 cfu/m^3^ (range 236–1076), 456 cfu/m^3^ (range 213–989), and 54 cfu/m^3^ (19–129) respectively. Comparing homes of cases versus controls, the median IMC (641 vs 477 cfu/m^3^), TBC (576 vs 428 cfu/m^3^) and TFC (66 vs 49 cfu/m^3^) were higher in homes of cases than controls (Table 3). The predominant bacterial agent found in homes of cases and controls were *Staphylococcus aureus* and *Staphylococcus epidermidis* (19% *and* 20%) while the dominant fungal agents were *Aspergillus niger* (23% *vs* 20%), and *Aspergillus fumigatus* (22% *vs* 11%). Significantly higher median bacterial and fungal diversity was found in homes of cases (*R* = 3.00; *H* = 1.04; *D* = 2.67 and *R* = 2.56; *H* = 0.82; *D* = 2.33) than homes of controls (*R* = 2.00; *H* = 0.64; *D* = 1.80 and *R* = 1.89; *H* = 0.55; *D* = 1.88) respectively (Fig. 2).

**Table 3:** Relative abundance of bacterial and fungal species in homes of cases and controls

Indoor Microbiome	Cases (n = 178); n (%)				Controls (n = 180); n (%)				p value
	GM (95% CI)	Median (IR)	Min	Max	GM (95% CI)	Median (IR)	Min	Max	
Total bacterial count (TBC) cfu/m^3^	575 (546–605)	544 (287)	237	989	428 (408–448)	414 (226)	213	732	< 0.001
* Staphylococcus aureus*	140 (127–153)	141 (47)	0	656	111 (95–125)	111 (147)	0	452	< 0.001
* Staphylococcus epidermidis*	69 (62–75)	70 (24)	0	328	54 (47–62)	55 (77)	0	226	< 0.001
* Streptococcus pneumoniae*	127 (113–140)	157 (193)	0	325	97 (83–111)	121 (168)	0	384	< 0.001
* Streptococcus pyogenes*	60 (53–67)	77 (95)	0	163	45 (32–52)	53 (83)	0	192	< 0.001
* Klebsiella aerogenes*	77 (64–91)	78 (168)	0	387	73 (57–89)	75 (127)	0	514	0.35
* Micrococcus spp.*	64 (50–77)	64 (160)	0	387	30 (19–41)	30 (0)	0	543	< 0.001
* Pseudomonas fluorescens*	38 (28–48)	38 (66)	0	400	16 (9–23)	16 (0)	0	270	< 0.001
* Unidentified colonies*	2 (2–3)	2 (2)	0	20	1 (0.4–1)	1 (0)	0	15	0.05
Total fungal count (TFC) cfu/m^3^	66 (63–69)	67 (35)	32	129	49 (46–52)	47 (25)	19	118	< 0.001
* Aspergillus niger*	19 (17–21)	19 (12)	0	73	12 (10–14)	12 (22)	0	70	< 0.001
* Aspergillus fumigatus*	9 (8–10)	9 (7)	0	29	5 (4–6)	5 (10)	0	38	< 0.001
* Penicillium spp.*	19 (16–22)	20 (26)	0	102	13 (11–16)	13 (22)	0	66	0.001
* Alternaria alternata*	6 (5–8)	6 (12)	0	65	4 (2–5)	4 (0)	0	45	0.001
* Fusarium oxysporum*	4 (3–6)	4 (0)	0	48	4 (3–6)	4 (0)	0	62	0.38
* Candida albicans*	7 (5–8)	7 (13)	0	74	4 (3–6)	4 (0)	0	38	0.03
* Cladosporium spp.*	3 (2–4)	3 (0)	0	38	5 (3–6)	5 (0)	0	73	0.54
* Unidentified colonies*	0.4 (0.2–0.7)	0 (0)	0	13	1 (1–2)	0 (0)	0	18	0.06
Indoor Microbial count (IMC) cfu/m^3^	641 (610–673)	606 (293)	283	1076	477 (455–498)	464 (246)	236	796	< 0.001

*IR* interquartile range, *GM* geometric mean, *Min* minimum, *Max* maximum, *cfu* colony forming unit

p value obtained using Mann–Whitney U test

IMC, TBC and TFC were positively and significantly (p < 0.001) correlated with indoor RH among cases (r = 0.56; r = 0.50; and r = 0.64), and controls (r = 0.47; r = 0.43; and r = 0.49) respectively, Table 4. The relationship between microbial diversity and indoor environmental conditions among cases revealed that bacterial richness and Simpson’s diversity (r = 0.39, r = 0.38) and fungal richness and diversity (r = 0.27, r = 0.29) correlated positively and significantly (p < 0.001) with indoor RH respectively (Table 4). A similar pattern was observed among controls.

**Table 4:** Spearman’s rank correlation coefficient between indoor microbial concentrations/diversity and environmental/household parameters among cases and controls

Indoor microbiome	Cases						Controls					
	Indoor temp. (°C)		Indoor RH (%)		Occupancy		Indoor temp. (°C)		Indoor RH (%)		Occupancy	
	rho	p value	rho	p value	rho	p value	rho	p value	rho	p value	rho	p value
Total bacterial count (TBC) cfu/m^3^	0.16*	0.04	0.50*	< 0.001	0.06	0.47	0.23*	0.002	0.43*	< 0.001	0.10	0.17
* Staphylococcus aureus*	0.07	0.34	0.08	0.30	0.03	0.73	0.06	0.42	0.21*	0.01	0.01	0.86
* Staphylococcus epidermidis*	0.08	0.29	0.07	0.35	0.01	0.96	0.09	0.26	0.16*	0.03	0.01	0.93
* Streptococcus pneumoniae*	0.06	0.41	0.12	0.10	0.01	0.86	0.05	0.52	0.16*	0.03	− 0.03	0.67
* Streptococcus pyogenes*	0.07	0.34	0.09	0.24	0.05	0.49	0.02	0.75	0.21*	0.01	− 0.03	0.65
* Klebsiella aerogenes*	0.09	0.23	0.27*	< 0.001	− 0.03	0.68	− 0.01	0.94	− 0.17	0.10	− 0.07	0.38
* Micrococcus spp.*	0.13	0.09	0.25*	0.001	0.04	0.63	0.13	0.08	0.03	0.71	0.12	0.11
* Pseudomonas fluorescens*	0.03	0.67	0.28*	< 0.001	− 0.02	0.77	− 0.03	0.73	− 0.10	0.19	0.05	0.53
* Unidentified colonies*	0.01	0.96	0.25	0.001	− 0.03	0.65	− 0.07	0.37	− 0.11	0.16	0.05	0.51
Total fungal count (TFC) cfu/m^3^	0.02	0.75	0.64*	< 0.001	− 0.01	0.90	0.13	0.09	0.49*	< 0.001	− 0.05	0.50
* Aspergillus niger*	0.05	0.51	0.16	0.04	0.15	0.05	− 0.02	0.81	0.08	0.27	− 0.10	0.18
* Aspergillus fumigatus*	0.04	0.57	0.15	0.05	− 0.08	0.30	0.12	0.11	0.27	< 0.001	0.01	0.99
* Penicillium spp.*	0.04	0.61	0.26*	< 0.001	0.15*	0.04	0.03	0.71	0.07	0.33	− 0.01	0.99
* Alternaria alternata*	0.07	0.33	0.10	0.17	− 0.03	0.70	0.01	0.88	0.14	0.06	− 0.01	0.95
* Fusarium oxysporum*	0.05	0.48	0.28*	< 0.001	0.01	0.87	0.07	0.34	0.09	0.22	− 0.11	0.15
* Candida albicans*	0.03	0.74	0.25*	0.001	− 0.02	0.82	0.01	0.95	0.08	0.29	0.16*	0.04
* Cladosporium spp.*	0.08	0.29	− 0.07	0.38	0.02	0.80	0.05	0.49	0.05	0.53	− 0.03	0.69
* Unidentified colonies*	0.08	0.28	− 0.02	0.82	− 0.05	0.48	0.01	0.87	− 0.02	0.82	0.08	0.31
Bacterial richness	0.16*	0.03	0.39*	< 0.001	0.06	0.43	0.10	0.18	0.08	0.28	− 0.04	0.57
Fungal richness	0.06	0.45	0.27*	< 0.001	0.04	0.58	0.05	0.52	0.32*	< 0.001	− 0.01	0.86
Bacterial diversity^†^	0.17*	0.03	0.38*	< 0.001	0.08	0.29	0.10	0.19	0.08	0.28	− 0.03	0.65
Fungal diversity^†^	0.07	0.37	0.29*	< 0.001	0.05	0.50	0.07	0.38	0.30*	< 0.001	0.01	0.86
IMC cfu/m^3^	0.16*	0.04	0.56*	< 0.001	0.05	0.55	0.22*	0.003	0.47*	< 0.001	0.10	0.19

*cfu/m*^*3*^ colony forming unit per meter cube, *rho* correlation coefficient; *RH* relative humidity, *temp.* temperature, *IMC* indoor microbial count

^†^Simpson’s diversity index; *P < 0.05

**Fig. 3:**
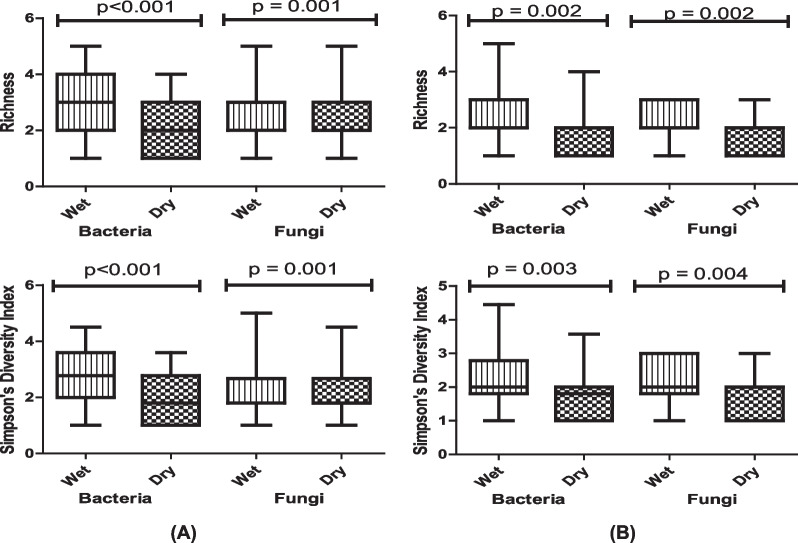
Seasonal variation in microbial richness and Simpson’s diversity index in homes of **A** cases and **B** controls. Each box and whisker plot shows: the minimum, first quartile (25th percentile), median (25th percentile), second quartile (75th percentile), and the maximum values; p value obtained using Mann–Whitney U test

Seasonal variation in the IMC, TBC and TFC stratified by case/control status is shown in Additional file 1: Table S2 (A and B). Comparing wet versus dry seasons, the median IMC (729 vs 484 cfu/m^3^), TBC (659 vs 418 cfu/m^3^) and TFC (70 vs 53 cfu/m^3^) in homes of cases were significantly different. Similar seasonal differences were observed among controls. In homes of cases, bacterial and fungal communities were significantly more diverse in the wet season (*R* = 3.00; *H* = 1.05; *D* = 2.78 and *R* = 3.00; *H* = 1.04; *D* = 2.67) than the dry season (*R* = 2.00; *H* = 0.64; *D* = 1.80 and *R* = 2.00; *H* = 0.63; *D* = 1.78). A similar pattern was observed among controls (Fig. 3A, B).

When adjusted by key covariates (Table 5), exposure to above median levels of lMC (OR = 2.67, 95% CI 1.44–4.97), TBC (OR = 2.51, 95% CI 1.36–4.65), TFC (OR = 2.75, 95% CI 1.54–4.89), bacterial richness (OR = 1.84, 95% CI 1.06–3.19), fungal richness (OR = 3.17, 95% CI 1.65–6.07), bacterial diversity (OR = 1.87, 95% CI 1.08–3.24) and fungal diversity (OR = 3.00, 95% CI 1.55–5.79), showed statistically significant associations with LRTI. Of note, the variable “presence of visible moulds in child’s sleeping area”, also showed a more than three-fold increased odds of LRTI (95% CI 1.36–15.02). Apart from these variables of interest, it is important to note that history of LRTI and non-exclusive breastfeeding were consistently associated with an increased risk of LRTI among under-five children in all models. Furthermore, home characteristics such as occupant density, house ownership, and use of dirty cooking fuel were associated with risk of LRTI among under-five children.

**Table 5:** Adjusted associations (aOR and 95% CI) for LRTI risk among under-five children using conditional multiple logistic regression model

Predictors	Crude OR (95% CI)	IMC	Indoor TBC	Indoor TFC	Bacterial richness (*R*)	Fungal richness (*R*)	Bacterial diversity^†^	Fungal diversity^†^	Visible mould in CSA
		aOR (95% CI)	aOR (95% CI)	aOR (95% CI)	aOR (95% CI)	aOR (95% CI)	aOR (95% CI)	aOR (95% CI)	aOR (95% CI)
High levels of exposures									
IMC	3.60 (2.24–5.79)*	2.67 (1.44–4.97)*							
Indoor TBC	3.30 (2.09–5.22)*		2.51 (1.36–4.65)*						
Indoor TFC	3.42 (2.12–5.50)*			2.75 (1.54–4.89)*					
Bacterial richness (*R*)	2.60 (1.66–4.06)*				1.84 (1.06–3.19)*				
Fungal richness (*R*)	3.77 (2.17–6.55)*					3.17 (1.65–6.07)*			
Bacterial diversity^†^	2.64 (1.69–4.11)*						1.87 (1.08–3.24)*		
Fungal diversity^†^	3.58 (2.05–6.23)*							3.00 (1.55–5.79)*	
Visible mould	5.18 (1.77–15.19)*								3.15 (1.36–15.02)*
Home characteristics									
Occupant density; > *5*	2.16 (1.30–3.59)*	2.77 (1.37–5.60)*	2.97 (1.47–6.00)*	2.71 (1.32–5.57)*	2.93 (1.46–5.88)*	2.89 (1.40–5.84)*	2.90 (1.45–5.82)*	2.99 (1.47–6.10)*	3.19 (1.59–6.42)*
Pet ownership; *any pet*	2.25 (1.42–3.58)*	1.53 (0.84–2.80)	1.46 (0.79–2.69)	1.80 (0.98–3.30)	1.75 (0.98–3.15)	1.77 (0.98–3.22)	1.75 (0.97–3.14)	1.75 (0.97–3.18)	1.79 (1.00–3.22)*
Any smoker; *yes*	2.11 (0.96–4.67)	2.05 (0.71–5.89)	2.02 (0.70–5.80)	1.81 (0.63–5.18)	2.42 (0.87–6.71)	2.08 (0.71–6.13)	2.42 (0.87–6.71)	2.06 (0.70–6.06)	2.67 (0.93–7.70)
Primary cooking fuel; *mixed*	2.25 (1.14–4.44)*	2.88 (1.15–7.22)*	2.90 (1.16–7.26)*	2.44 (1.00–5.97)*	2.56 (1.04–6.33)*	2.67 (1.07–6.66)*	2.55 (1.03–6.31)*	2.68 (1.08–6.66)*	2.90 (1.18–7.12)*
House ownership; *rented*	2.17 (1.33–3.56)*	2.51 (1.27–4.97)*	2.57 (1.29–5.09)*	2.43 (1.22–4.85)*	2.64 (1.35–5.17)*	2.68 (1.35–5.34)*	2.63 (1.34–5.14)*	2.68 (1.35–5.31)*	2.66 (1.38–5.12)*
Wet season	1.75 (1.06–2.89)*	1.44 (0.70–2.99)	1.45 (0.70–2.98)	1.05 (0.52–2.12)	1.11 (0.56–2.20)	1.00 (0.50–1.99)	1.12 (0.56–2.23)	1.03 (0.52–2.05)	1.00 (0.51–1.96)
Child’s characteristics									
History of LRTI; yes	4.09 (1.35–12.38)*	11.78 (1.15–21.22)*	11.69 (1.16–20.16)*	13.73 (1.32–24.35)*	12.06 (1.31–20.05)*	9.87 (1.08–19.40)*	12.09 (1.31–19.24)*	9.79 (1.07–17–53)*	11.31 (1.27–21.86)*
No. of siblings; ≥ *1*	2.51 (1.16–5.43)*	2.58 (1.05–6.33)*	2.63 (1.07–6.45)*	2.35 (0.94–5.89)	2.56 (1.05–6.25)*	2.65 (1.06–6.62)*	2.56 (1.05–6.25)*	2.67 (1.08–6.60)*	2.42 (1.00–5.91)*
Non-exclusive breastfeeding	2.45 (1.41–4.25)*	2.18 (1.08–4.38)*	2.18 (1.09–4.35)*	2.31 (1.13–4.74)*	2.10 (1.06–4.15)*	2.29 (1.13–4.67)*	2.10 (1.06–4.15)*	2.31 (1.13–4.70)*	2.01 (1.02–3.95)*
Pseudo R^2^		0.23	0.22	0.23	0.20	0.22	0.20	0.23	0.20

Exposures > median value is considered as high levels of exposures

All multivariate models were adjusted for age, sex, geographical location, occupancy, pet ownership, ETS, season, house ownership, history of LRTI, number of siblings under-five and breastfeeding status

*OR* odds ratio, *aOR* adjusted odds ratio, *CSA* child’s sleeping area, *CI* confidence interval, *TBC* total bacterial count, *IMC* indoor microbial count, *TFC* total fungal count

^*^p < 0.05

^†^Simpson diversity index (*D*) was used as a measure of microbial diversity in the model

## Discussion

Our study found a significantly increased risk of LRTI among under-five children in the higher categories of indoor microbial exposures, after adjusting for critical covariates such as housing and child characteristics. Subject to exposure variable, there was a 2–threefold statistically significant increase in risk of LRTI among under-five children, with the indices for exposure to fungal aerosols being at the higher end of this range.

We found that quantitative indices of exposure to indoor microbial aerosols such as IMC, TBC, and TFC, and bacterial and fungal diversity measures were positively associated with LRTI risk among under-five children. This finding corroborates the few available studies that employed similar methods [19, 30]. A recent systematic review and meta-analysis [16] reported exposure to high concentrations of indoor microbial aerosols to be associated with an increased risk of LRTI among under-five children (pooled OR = 1.20; 95% CI 1.11–1.33), with a higher risk from exposure to TFC (pooled OR = 1.27; 95% CI 1.13–1.33). This suggest that possible exposure to indoor microbial aerosols should be considered in the development of preventive measures and treatment options for childhood LRTI. A study carried out among students in China contradicts our findings and reported no association between overall microbial richness and respiratory infection (OR = 1.00; 95% CI 0.83–1.21), but a link between abundance of several microbial genera in the Gammaproteobacteria class and occurrence of respiratory infection [31].

The bacterial genera observed in this study commonly occur in the indoor and outdoor environment but could become opportunistic in immunocompromised individuals [32]. This implies that microorganisms that usually inhabit a particular indoor environment could become infectious or pathogenic when the immune system of the host becomes compromised especially among under-five children with immature immune response, thereby leading to unhealthy conditions such as LRTIs. As obtained in the current study, a recent clinical study reported *Streptococcus pneumoniae* as the leading pathogen of LRTI even with the introduction of 13-valent pneumococcal conjugate vaccine [33], followed by *Hemophilus influenza* and *Klebsiella pneumonia* [34]. Also, a similar study in Nigeria, reported *Klebsiella pneumoniae* as the most detected pathogen [35] in LRTIs*.* This therefore confirms that bacteria play a major role in the aetiology of LRTI among under-five children. The fungal genera identified were similar to the submission by Ana et al. [20], who found *Aspergillus spp* to be the dominant fungal species in home of under-five children with and without acute respiratory infections. Furthermore, we observed a significant difference in the microbial diversity during the wet compared to dry seasons among cases and controls which may suggest a seasonal variation. Even though indoor temperature and RH were found to be significantly correlated with indoor microbial concentrations, it does not necessarily mean that meteorological variables exert a direct effect on the microbial community.

Proxy assessment of microbial exposure such as presence of visible moulds on a variety of surfaces in the house, often used in studies [18, 30], were found to result in similar associations with LRTI as the quantitative measures of microbial exposures employed in the current study, but the quantitative measures provide additional useful evidence of the types and diversity of the microbial community which cannot be obtained using the proxy measures. We found a greater risk of LRTI among under-five children with exposure to fungal aerosols. Stark et al. [19], in their study found a significantly increased risk of LRTI in infants with exposure to higher in-home fungal concentrations (RR = 1.86, 95% CI 1.21–2.88). In addition, they reported presence of visible mould growth to be an independent predictor of LRTI in the first year of life (OR = 1.34, 95% CI 0.99–1.82) [19]. In support of our findings, a case–control study carried out in New Zealand among under-five children to investigate the dose–response association of objectively assessed housing quality measures, particularly the presence of visible moulds presented as the damp-mold index (DMI) and hospitalization with acute respiratory infections (ARI) showed a significant adjusted dose–response relationship (aOR = 1.15; 95% CI 1.02–1.30) [18]. Possible biological mechanism could be that prolonged exposure to aerosolized fungal components mainly target the respiratory and nervous system causing specific pathological changes in the host characterized by inflammation of the mucosal lining of the airways [36]. Relevant studies both in vitro and in vivo have demonstrated that repeated activation of immune responses and inflammation from fungal exposures may contribute to inflammation-related diseases, and the resulting inflamed mucosal tissue may provide a diminished barrier to respiratory infections [37].

The high microbial concentrations and diversity recorded in homes of under-five children with LRTI could be attributable to the high occupant density, reduced ventilation and high indoor RH. A modest positive correlation was recorded between indoor microbial exposure indices and indoor RH which was corroborated with previous reports by Frankel et al. [38], who found that indoor RH correlated positively with indoor fungal exposure (r = 0.32, p = 0.002). Increased relative humidity contributes to microbial survival [39], and antigenic potential from fungi [40], and can better facilitate the direct-contact transfer of microorganisms [41]. Even though the microbial concentrations and diversity in the current study was not significantly correlated with occupant density, a number of studies have established that occupancy is associated with increased microbial concentrations, and diversity and abundance of human-associated microbes in indoor environment [42, 43]. This incongruity is probably due to the nature of the environment where these studies were carried out.

Also, we observed that child characteristics such as history of LRTI, > 1 under five sibling and non-exclusive breastfeeding, and household characteristics such as house ownership, high occupant density, and use of dirty cooking fuels in the household were independently associated with LRTI risk among under-five children which are consistent with previous epidemiological studies [5, 44, 45]. Environmental Tobacco smoking (ETS) was not found to be significantly associated with LRTI risk but was a potential risk factor. This is possibly because smoking is not encouraged in the environment where the current study was carried out, therefore only very few individuals reported their smoking status. Notably, even after adjusting for these important factors, all microbial exposure indices remained significantly linked to LRTI risk among under-five children.

The median age of subjects recorded in the current study suggests that most of the under-five children with LRTI were below 12 months of age. This is similar to the report by Ahmed et al., in their study to assess the risk factors for acute lower respiratory tract infections (ALRTI) among hospitalized children under-five in Northern Nigeria. They reported children between 2 to 12 months of age to have accounted for 56.0% of hospitalization due to ALRTI [46]. Studies in other developing countries such as Ethiopia [47] and Rwanda [48] also showed similar findings. The preponderance of male sex among under-five children with LRTI observed in the current study is also similar to previous studies on LRTI among under-five children [46, 48].

A major strength of the study was the objective measure of microbial exposure from culture-dependent methods which provided detailed information of the microbial concentrations and diversity, as compared to other studies that employed proxy measures. Although culture-dependent method has been reported to underestimate the types of microorganisms observed by not accounting for non-culturable microbes, it has been proven to provide useful information in the study of microbial diversity [49]. The assessment of microbial diversity using species richness, Shannon and Simpson Diversity Indices was a unique step that gave insight into the microbial community and seasonal pattern in this environment. In addition, the diagnosis of LRTI based on chest radiography was a major strength as this help minimize the risk of misclassification.

A limitation of the study was the use of respiratory signs and symptoms to define (absence of) LRTI among community controls which could have introduced some outcome definition bias. This was strongly minimized by actively engaging the same doctor that assessed cases in the assessment of the community controls. The use of respiratory signs and symptoms in defining LRTI among under-five children is a common practice especially in sub–Saharan Africa (SSA) [20, 44, 50] and we strongly believe it has no impact on the validity and generalizability of our results. The inability to assess the siblings of cases on episodes of LRTI is another limitation of the study as this information could have further supported the association with exposure to diverse microbial aerosols. It is possible that households especially for cases may have changed the practices before home visitation, therefore the observed measured concentrations could be lower compared to when the children were infected, resulting in underestimation of the risk associated with microbial exposure. However, this was minimized by a short lag from recruitment into the study, and the home survey.

## Conclusions

This study provides comprehensive epidemiological evidence of the microbial concentration and diversity in residential environments in sub-Saharan Africa in relation to LRTI among under-five children. This study suggests that exposure to indoor microbial aerosols is independently associated with LRTI risk among under-five children. The increased risk was most pronounced for fungal aerosols. Therefore, indoor microbial exposure-tailored intervention should be considered in the management of LRTI among children under the age of five years.

## Supplementary Information

**Additional file 1. Table S1**: Indoor Air Microbiome and ARTI study. **Table S2**: Seasonal variation in the relative abundance of Bacterial and Fungal genera stratified by case/control status.

## Data Availability

All data generated or analysed during this study are included in this published article and its additional files. Individuals interested in obtaining the data required to replicate the results should contact Dr. Adekunle Fakunle; fakunz@yahoo.com.

## Abbreviations

LRTI: Lower respiratory tract infections
IMC: Indoor microbial count
TBC: Total bacterial count
TFC: Total fungal count
H: Shannon diversity index
D: Simpson diversity index
OR: Odds ratio
aOR: Adjusted odds ratio
95%CI: 95% Confidence interval
RH: Relative humidity
p: p value
r: Correlation coefficient
IQR: Interquartile range
