# Supplementary material for: Indoor bacterial and fungal aerosols as predictors of lower respiratory tract infections among under-five children in Ibadan, Nigeria
Source: BMC Pulm Med. 2022 Dec 9;22:471. doi: 10.1186/s12890-022-02271-w (PMC9733100; doi:10.1186/s12890-022-02271-w)
Supplement: Supplementary file 1 — Additional file 1. Table S1: Indoor Air Microbiome and ARTI study. Table S2: Seasonal variation in the relative abundance of Bacterial and Fungal genera stratified by case/control status. [file 12890_2022_2271_MOESM1_ESM.docx]

**SUPLEMENTARY MATERIAL**

# Table S1: Indoor Air Microbiome and ARTI study

# Clinical Proforma

| **Date:____/____/_______**  **Day Month Year** | **Study Identification No.** |  |  | **-** |  |  | **-** |  |  |  |
| --- | --- | --- | --- | --- | --- | --- | --- | --- | --- | --- |

| 1. Child’s name | ________________ , _________________, _________________  First Middle Surname |
| --- | --- |
| 2. Hospital number | _____________________________________ |
| 3. Date of Birth | Day_____/Month_____/ Year______; Age _______**month** |
| 4. Sex | 🞎_1_ Male 🞎_2_ Female |
| 5. i. Breastfeeding   1. Duration of breastfeeding | _0_ Exclusive  _1_ Not exclusive  _2_ Never breastfed  _________________**months** |
| 6. i. Immunization  (**verified by immunization card**)  ii. Specify vaccines received: | _0_ Completed immunization  _1_ Ongoing  _2_ Not completed  _3_ Not immunized |
|  | _0_ OPV_0 4_ BCG _8_ PCV1 _12_ IPV  _1_ OPV_1 5_ Penta1 _9_ PCV2 _13_ Yellow Fever  _2_ OPV_2 6_ Penta2 _10_ PCV3  _3_ OPV_3 7_ Penta3 _11_ Measles |
| 7. Anthropometry | Length: _**____________cm**; Weight: _________________**kg** |
| 8. Clinical measurements | Temperature _______**°C**; Respiratory rate ________**breath/min** |
| 9. Any respiratory symptoms? | _0_ No, [**IF NO MOVE TO Q. 10**]  _1_ Yes |
| 1. Cough | _0_ No,  _1_ Yes |
| 1. Fever. | _0_ No,  _1_ Yes |
| 1. Wheezing | _0_ No,  _1_ Yes |
| 1. Difficulty in breathing. | _0_ No,  _1_ Yes |
| 10. Any respiratory signs? | _0_ No, [**IF NOT GO TO Q 11**]  _1_ Yes |
| 1. Fast breathing. | _0_ No,  _1_ Yes |
| 1. Intercostal recession | _0_ No,  _1_ Yes |
| 1. Lower chest wall indrawing | _0_ No,  _1_ Yes |
| 1. Respiratory crackles | _0_ No,  _1_ Yes |
| 11. Type [**SELECT ONLY ONE OPTION**] | _1_ Non severe pneumonia  _2_ Severe pneumonia.  _3_ Pleural effusion.  _4_ None of the above |
| 12. Other diagnosis [**SELECT AS MANY AS APPLICABLE**] | _1_ Malaria  _2_ Sepsis.  _3_ Meningitis.  _4_ Diarrhoeal disease.  _5_ Others (specify): _____________________________________ |
| 13. CXR Findings [**SELECT AS MANY AS APPLICABLE**] | _1_ Uniform Consolidation  _2_ Non-uniform infiltrates  _3_ Pleural effusion  _4_ No Consolidation  ____________________________________ |
| 14. Pleural Aspirate M/C/S | Lymphocyte_____________ Neutrophils ______________  Eosinophils _____________Basophil _______________ |
| 15. Blood culture | Organisms: 1. ______________________________  2. ______________________________  3. ______________________________  Sensitivity Pattern: _______________________________________________________________________________________________________ |
| 16. i. Outcome  ii. Duration of hospital stay   1. Duration of use of oxygen | _1_ Discharged  _2_ Discharged against medical advise  _3_ Dead  ________________________**days**  ________________________**days** |

**Table S2: Seasonal variation in the relative abundance of Bacterial and Fungal genera stratified by case/control status**

1. **Cases**

| **Indoor Microbiome** | **Wet (n = 107)** | | | | **Dry (n = 71)** | | | | **p-value** |
| --- | --- | --- | --- | --- | --- | --- | --- | --- | --- |
|  | **GM (95% CI)** | **Median (IR)** | **Min** | **Max** | **GM (95% CI)** | **Median (IR)** | **Min** | **Max** |  |
| **Total bacterial count (TBC) cfu/m^3^** | 659 (621-697) | 657 (354) | 265 | 989 | 449 (421-477) | 418 (165) | 237 | 702 | <0.001 |
| *Staphylococcus aureus* | 149 (132-167) | 142 (32) | 0 | 656 | 126 (106-147) | 133 (185) | 0 | 317 | 0.27 |
| *Staphylococcus epidermidis* | 74 (65-83) | 71 (17) | 0 | 328 | 61 (50-71) | 67 (90) | 0 | 159 | 0.16 |
| *Streptococcus pneumoniae* | 142 (126-157) | 165 (71) | 0 | 283 | 104 (80-128) | 133 (190) | 0 | 325 | 0.03 |
| *Streptococcus pyogenes* | 66 (58-74) | 79 (95) | 0 | 142 | 51 (39-62) | 67 (94) | 0 | 163 | 0.07 |
| *Klebsiella aerogenes* | 95 (79-112) | 108 (179) | 0 | 300 | 50 (28-72) | 53 (95) | 0 | 387 | <0.001 |
| *Micrococcus spp.* | 81 (62-100) | 82 (190) | 0 | 345 | 38 (21-56) | 40 (75) | 0 | 387 | 0.002 |
| *Pseudomonas fluorescens* | 51 (36-67) | 51 (100) | 0 | 400 | 17 (8-26) | 19 (0) | 0 | 160 | 0.02 |
| *Unidentified colonies* | 3 (2-4) | 4 (5) | 0 | 20 | 2 (0.5-2.5) | 2 (0) | 0 | 20 | 0.08 |
|  |  |  |  |  |  |  |  |  |  |
| **Total fungal count (TFC) cfu/m^3^** | 70 (66-74) | 69 (32) | 35 | 129 | 60 (56-65) | 53 (23) | 32 | 112 | 0.003 |
| *Aspergillus niger* | 19 (16-21) | 19 (13) | 0 | 51 | 18 (15-22) | 19 (25) | 0 | 73 | 0.66 |
| *Aspergillus fumigatus* | 9 (8-10) | 9 (7) | 0 | 25 | 9 (7-10) | 9 (13) | 0 | 29 | 0.86 |
| *Penicillium spp.* | 20 (17-23) | 20 (29) | 0 | 78 | 18 (13-22) | 17 (25) | 0 | 102 | 0.07 |
| *Alternaria alternata* | 6 (4-8) | 6 (12) | 0 | 50 | 6 (3-10) | 6 (12) | 0 | 65 | 0.39 |
| *Fusarium oxysporum* | 5 (4-7) | 6 (10) | 0 | 48 | 3 (1-5) | 3 (5) | 0 | 45 | 0.06 |
| *Candida albicans* | 8 (6-11) | 8 (15) | 0 | 74 | 4 (2-6) | 5 (11) | 0 | 30 | 0.03 |
| *Cladosporium spp.* | 3 (2-4) | 3 (8) | 0 | 38 | 3 (1-4) | 3 (6) | 0 | 30 | 0.87 |
| *Unidentified colonies* | 0.5 (0.2-0.8) | 0 (0) | 0 | 13 | 0.4 (0.1-0.8) | 0 (0) | 0 | 10 | 0.73 |
| **Indoor Microbial count (IMC) cfu/m^3^** | 729 (689-769) | 728 (365) | 341 | 1076 | 509 (480-539) | 484 (180) | 283 | 767 | <0.001 |

IR – Interquartile range Max – Maximum

GM – Geometric mean cfu – Colony forming unit

Min – Minimum

1. **Controls**

| **Indoor Microbiome** | **Wet (n = 89)** | | | | **Dry (n = 89)** | | | | **p-value** |
| --- | --- | --- | --- | --- | --- | --- | --- | --- | --- |
|  | **GM (95% CI)** | **Median (IR)** | **Min** | **Max** | **GM (95% CI)** | **Median (IR)** | **Min** | **Max** |  |
| **Total bacterial count (TBC) cfu/m^3^** | 500 (473-527) | 467 (231) | 213 | 732 | 356 (334-377) | 321 (147) | 213 | 587 | <0.001 |
| *Staphylococcus aureus* | 132 (111-153) | 127 (100) | 0 | 452 | 89 (70-109) | 91 (144) | 0 | 391 | 0.01 |
| *Staphylococcus epidermidis* | 65 (54-75) | 60 (49) | 0 | 226 | 44 (34-54) | 33 (74) | 0 | 196 | 0.02 |
| *Streptococcus pneumoniae* | 113 (94-132) | 133 (168) | 0 | 384 | 81 (61-101) | 75 (168) | 0 | 379 | 0.03 |
| *Streptococcus pyogenes* | 56 (47-66) | 67 (85) | 0 | 192 | 34 (24-44) | 31 (72) | 0 | 189 | 0.001 |
| *Klebsiella aerogenes* | 83 (60-107) | 71 (152) | 0 | 514 | 63 (42-84) | 53 (109) | 0 | 416 | 0.15 |
| *Micrococcus spp.* | 40 (21-59) | 28 (0) | 0 | 543 | 20 (9-31) | 12 (0) | 0 | 247 | 0.31 |
| *Pseudomonas fluorescens* | 10 (3-18) | 5 (0) | 0 | 270 | 21 (10-34) | 14 (0) | 0 | 267 | 0.17 |
| *Unidentified colonies* | 1 (1-2) | 1 (0) | 0 | 15 | 1 (1-2) | 1 (0) | 0 | 14 | 0.07 |
|  |  |  |  |  |  |  |  |  |  |
| **Total fungal count (TFC) cfu/m^3^** | 54 (50-59) | 51 (27) | 22 | 118 | 43 (40-47) | 41 (28) | 19 | 98 | 0.002 |
| *Aspergillus niger* | 12 (8-16) | 12 (22) | 0 | 70 | 12 (9-15) | 12 (24) | 0 | 59 | 0.78 |
| *Aspergillus fumigatus* | 6 (4-8) | 6 (13) | 0 | 38 | 4 (2-5) | 4 (1) | 0 | 27 | 0.08 |
| *Penicillium spp.* | 13 (10-17) | 12 (23) | 0 | 66 | 14 (10-17) | 13 (23) | 0 | 66 | 0.79 |
| *Alternaria alternata* | 5 (3-7) | 4 (0) | 0 | 45 | 3 (1-4) | 2 (0) | 0 | 27 | 0.18 |
| *Fusarium oxysporum* | 6 (3-8) | 6 (6) | 0 | 62 | 2 (1-4) | 2 (0) | 0 | 38 | 0.02 |
| *Candida albicans* | 5 (3-7) | 5 (0) | 0 | 38 | 4 (2-6) | 4 (0) | 0 | 37 | 0.70 |
| *Cladosporium spp.* | 6 (3-9) | 5 (0) | 0 | 73 | 3 (1-5) | 2 (0) | 0 | 40 | 0.13 |
| *Unidentified colonies* | 2 (1-3) | 2 (0) | 0 | 18 | 2 (1-3) | 2 (0) | 0 | 17 | 0.95 |
| **Indoor Microbial count (IMC) cfu/m^3^** | 555 (526-583) | 531 (254) | 237 | 796 | 399 (375-423) | 364 (164) | 236 | 676 | <0.001 |

IR – Interquartile range

GM – Geometric mean

Min – Minimum

Max – Maximum

cfu – Colony forming unit
